# Constitutive Activation of p62/Sequestosome-1-Mediated Proteaphagy Regulates Proteolysis and Impairs Cell Death in Bortezomib-Resistant Mantle Cell Lymphoma

**DOI:** 10.3390/cancers14040923

**Published:** 2022-02-12

**Authors:** Grégoire Quinet, Wendy Xolalpa, Diana Reyes-Garau, Núria Profitós-Pelejà, Mikel Azkargorta, Laurie Ceccato, Maria Gonzalez-Santamarta, Maria Marsal, Jordi Andilla, Fabienne Aillet, Francesc Bosch, Felix Elortza, Pablo Loza-Alvarez, Brigitte Sola, Olivier Coux, Rune Matthiesen, Gaël Roué, Manuel S. Rodriguez

**Affiliations:** 1 Laboratoire de Chimie de Coordination (LCC) CNRS-UPR8241, UPS, 31400 Toulouse, France; gregoire.quinet@mfr-eyzinpinet.org (G.Q.); laurie.bonnafe@evotec.com (L.C.); Maria.GONZALEZ@lcc-toulouse.fr (M.G.-S.); 2 Proteomics Unit, CIC bioGUNE, Parque Tecnológico de Bizkaia, 48160 Derio, Spain; wendy.xolalpa@ibt.unam.mx (W.X.); fabienne.aillet@clinact.com (F.A.); 3 Lymphoma Translational Group, UBIRed, Josep Carreras Leukaemia Research Institute, 08916 Badalona, Spain; diana.reyes@upf.edu (D.R.-G.); nprofitos@carrerasresearch.org (N.P.-P.); 4 Proteomics Platform CICbioGUNE, Basque Research and Technology Alliance (BRTA), CIBERehd, ProteoRed-ISCIII, Parque Tecnológico de Bizkaia, 31400 Derio, Spain; mazkargorta@cicbiogune.es (M.A.); felortza@cicbiogune.es (F.E.); 5 ICFO-Institut de Ciencies Fotoniques, The Barcelona Institute of Science and Technology, 08860 Castelldefels, Spain; Maria.Marsal@icfo.eu (M.M.); jordi.andilla@icfo.eu (J.A.); pablo.loza@icfo.eu (P.L.-A.); 6 Laboratory of Experimental Hematology, Department of Hematology, Vall d’Hebron Institute of Oncology, Vall d’Hebron University Hospital, 08035 Barcelona, Spain; fbosch@vhio.net; 7 INSERM UMR1245, Unicaen, 14000 Caen, France; brigitte.sola@unicaen.fr; 8 Centre de Recherche de Biologie Cellulaire de Montpellier (CRBM) CNRS-UMR 5237, Université de Montpellier, 34293 Montpellier, France; olivier.coux@crbm.cnrs.fr; 9 Computational and Experimental Biology Group, CEDOC-Chronic Diseases Research Center, Faculdade de Ciências Médicas, Universidade Nova de Lisboa, 1150-082 Lisboa, Portugal; rune.matthiesen@nms.unl.pt

**Keywords:** apoptosis, autophagy, proteasome inhibitor, TUBEs, ubiquitin proteome, verteporfin

## Abstract

**Simple Summary:**

To decipher the molecular mechanism underlying the resistance of a significant fraction of mantle cell lymphoma (MCL) patients to the first-in-class proteasome inhibitor bortezomib (BTZ), we have characterized the ubiquitin-related proteome (i.e., ubiquitome) of a set of MCL cell lines with different degrees of sensitivity to the drug by coupling a tandem ubiquitin-binding entity (TUBE) approach to mass spectrometry, followed by phenotypic and functional validations in both in vitro and in vivo models of MCL. We identified an enrichment of autophagy–lysosome system (ALS) components in BTZ-resistant cells, which was associated with constitutive intracellular inactivation of proteasome subunits by a process called proteaphagy. Blockade of this phenomenon by the pharmacological or genetic inactivation of the autophagy receptor p62/SQSTM1 reactivated normal proteasomal activity and restored the BTZ antitumor effect in in vitro and in vivo models of BTZ resistance.

**Abstract:**

Protein ubiquitylation coordinates crucial cellular events in physiological and pathological conditions. A comparative analysis of the ubiquitin proteome from bortezomib (BTZ)-sensitive and BTZ-resistant mantle cell lymphoma (MCL) revealed an enrichment of the autophagy–lysosome system (ALS) in BTZ-resistant cells. Pharmacological inhibition of autophagy at the level of lysosome-fusion revealed a constitutive activation of proteaphagy and accumulation of proteasome subunits within autophagosomes in different MCL cell lines with acquired or natural resistance to BTZ. Inhibition of the autophagy receptor p62/SQSTM1 upon verteporfin (VTP) treatment disrupted proteaphagosome assembly, reduced co-localization of proteasome subunits with autophagy markers and negatively impacted proteasome activity. Finally, the silencing or pharmacological inhibition of p62 restored the apoptosis threshold at physiological levels in BTZ-resistant cells both in vitro and in vivo. In total, these results demonstrate for the first time a proteolytic switch from the ubiquitin–proteasome system (UPS) to ALS in B-cell lymphoma refractory to proteasome inhibition, pointing out a crucial role for proteaphagy in this phenomenon and paving the way for the design of alternative therapeutic venues in treatment-resistant tumors.

## 1. Introduction

Mantle cell lymphoma (MCL) is a rare B-cell lymphoma that represents 5–10% of all non-Hodgkin lymphomas, with an incidence of 0.8 cases per 100,000 persons [1]. It develops primarily among elderly individuals with a median age of approximately 67 years and a male-to-female ratio of 2–3:1. The clinical evolution is usually very aggressive, and despite overall response rates above 70% with first-line standard immunochemotherapeutic schemes, few patients can be cured [2,3]. Proteasome inhibition was demonstrated to be an efficient strategy for relapsed or refractory (R/R) MCL patients. Bortezomib (BTZ) was the first proteasome inhibitor (PI) approved as a second-line treatment in this disease. However, more than half of patients display either an innate resistance or develop resistance along the course of the treatment. The development of new PIs (carfilzomib, ixazomib) has not solved the problem [4]. BTZ inhibits the proteasome by targeting the β5 subunit of the 20S core particle (CP), thereby impairing degradation of intracellular proteins, including crucial factors regulating cell cycle, tumor progression and apoptosis [5]. Resistance to BTZ treatment appears in patients and has been extensively investigated in hematologic cancers over the last decade [6]. Point mutations in the β5 catalytic subunits of the proteasome occasionally explain BTZ resistance [7,8]. Nevertheless, such mutations are not always found in BTZ-resistant cells, indicating that alternative mechanisms must support drug refractoriness. These alternative mechanisms include dysfunction of oxidative and endoplasmic reticulum stress [9], activation of the cytoprotective arm of the unfolded protein response [10] or re-activation of a plasmacytic differentiation program [11]. Consistently, pharmacological modulation of these pathways can partially re-sensitize resistant MCL cells to BTZ [7].

The ubiquitin–proteasome system (UPS) and the autophagy–lysosome system (ALS) constitute the main intracellular proteolytic pathways in eukaryotic cells [12,13]. Proteasomes are large protein complexes that ensure the selective degradation of cytosolic and nuclear proteins in response to a vast diversity of specific signals. The best understood proteasome form is the 26S proteasome, which degrades ubiquitylated proteins. It is formed by the 20S CP carrying the proteolytic activities and the 19S regulatory particle (RP) that recruits the target proteins and injects them into the CP. The 20S CP possesses three catalytic subunits, β1, β2 and β5, with caspase-like, trypsin-like and chymotrypsin-like peptidase activities, respectively [14,15].

Ubiquitin (Ub) and ubiquitin-like proteins (UbLs) are also implicated in ALS-mediated proteolysis. In particular, members of the LC3/GABARAP family are involved in the formation of lipidic membranes, called phagophores, that engulf targeted cargos. Subsequently, they fuse with lysosomes to drive substrate degradation [12,13]. Distinct selective autophagy pathways have been reported and named according to the type of substrate that is embedded and degraded, such as mitophagy (mitochondria) or proteaphagy (proteasome). Recently, Marshall et al. showed in *A. thaliana* that proteaphagy is activated under proteasome inhibition and nutrient starvation [16,17]. Proteaphagy has also been described in mammalian cells [18,19,20]. These results revealed a novel level of interaction between UPS and ALS. 

To analyze the molecular impact of BTZ resistance in MCL, we investigated the Ub-dependent proteome of MCL cells. To this end, tandem ubiquitin-binding entities (TUBEs) coupled with mass spectrometry (MS) analysis were performed [21,22,23]. Here we report the comparison of TUBEs-associated Ub-proteomes from MCL cell lines refractory (ZBR) or responsive (Z-138) to BTZ. Similar changes were obtained by comparing other sensitive and resistant MCL cells, highlighting the relevance of our observations. Our results show that ALS compensates a defective UPS found in BTZ-resistant cells by a permanently activated proteaphagy and that autophagy receptor p62 fulfills a key role in the assembly of proteaphagosomes. We further demonstrate that silencing or pharmacological inhibition of p62 reactivates apoptosis signalling in vitro and in vivo in MCL tumors with intrinsic or acquired resistance to BTZ.

## 2. Results

### 2.1. Reduction of UPS Is Compensated by ALS Factors in BTZ-Resistant MCL Cells 

**Figure 1:**
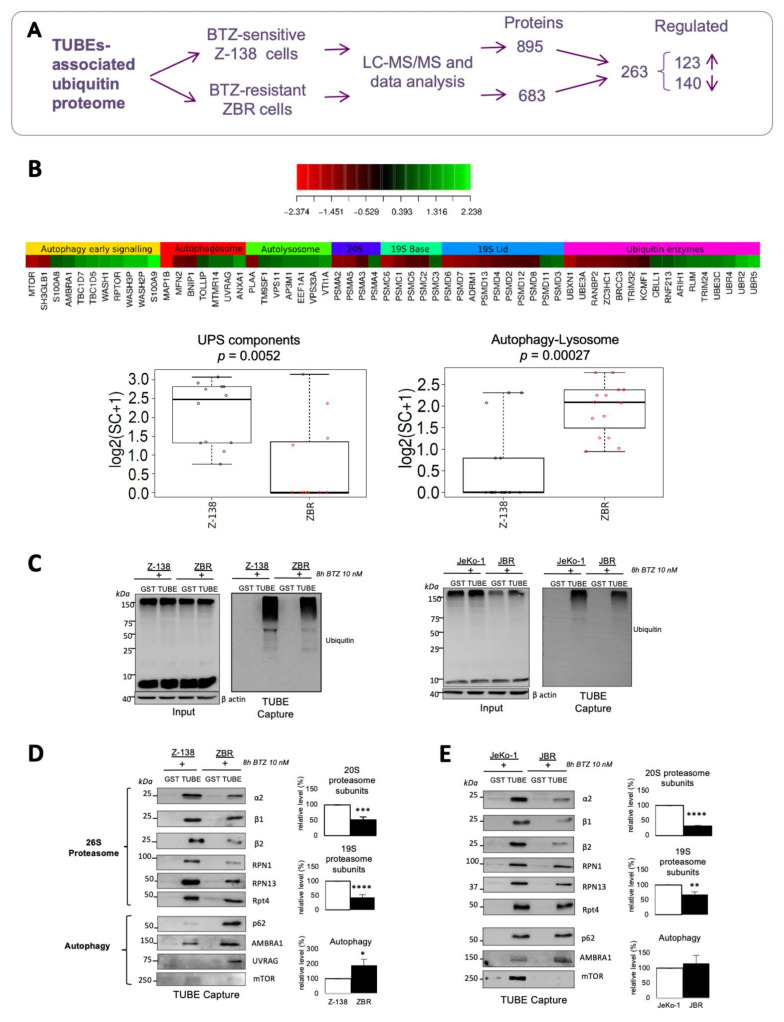
Analysis of the TUBEs-associated Ub proteome of BTZ-resistant MCL cells. (**A**) Scheme of the strategy used to isolate and compare the Ub proteome of Z-138 and ZBR MCL cells. (**B**) Heat map and boxplots showing significantly functionally enriched UPS and ALS categories in BTZ-resistant ZBR cells compared to the parental Z-138 cell line. Red = low enrichment; green = high enrichment. (**C**) Ubiquitylation pattern in Z-138 vs. ZBR and JeKo-1 vs. JBR cells. Ubiquitylated proteins were captured using TUBEs from Z-138/ZBR (**D**) and JeKo-1/JBR (**E**). GST was used as a control. Indicated fractions were analyzed by WB with the indicated antibodies (Abs). The densities of proteins calculated for 19S, 20S and autophagy are the means of all single values. Quantifications were performed using ImageJ software (*n* ≥ 3; mean ± SD; two-tailed Student’s *t*-test, * *p* < 0.05, ** *p* < 0.01, *** *p* < 0.001, **** *p* < 0.0001). White and black columns refer to parental and BTZ-resistant derived cell lines, respectively. *n* represents the mean of three replicates for each of the subunits analysed that were integrated into 20S or 19S subunits.

The biological impact of proteasome inhibition in MCL leads to the accumulation of ubiquitylated proteins, inducing proteotoxic stress and affecting crucial signaling pathways [8,24]. We hypothesized that these accumulated proteins could potentially be implicated in the response or resistance to BTZ. To acquire insight into the nature of these proteins, we compared the Ub-proteome isolated from a representative, BTZ-sensitive MCL cell line (Z138) and from its BTZ-resistant derived sub-clone (ZBR) [10]. We used TUBEs previously shown to be efficient for purification of Ub-proteins [22,25,26,27]. We identified 895 proteins that were specifically bound to TUBEs in Z-138 cells and 683 in ZBR cells (Figure 1A and Figure S1A–E). Of these proteins, 263 were reduced or enriched in these cell lines and were retained for analysis (Table S1). Ingenuity pathway analysis (IPA) showed that protein ubiquitylation, phagosome maturation and unfolding protein response were in the top five most represented pathways (Figure S1F). Gene ontology (GO) analysis was used to obtain an integrated heatmap view of the simultaneous Ub-regulated processes occurring in both BTZ-resistant and -sensitive cells (Figure S2). Crucial differences were observed among the 60 proteins of the UPS and ALS. In particular, proteasome subunits were reduced while components of the ALS were enriched in ZBR compared to Z-138 cells (Figure 1B and Figure S3A). Other changes in the UPS included Ub-ligases, de-ubiquitylating enzymes and total protein ubiquitylation (Figure 1B,C).

We confirmed the reduced levels of proteasome subunits (α and β subunits of the CP, base and lid subunits of the 19S complex) associated with the Ub-proteome in ZBR cells compared to Z-138 cells by western blot (WB) using specific antibodies (Figure 1D and Figure S3B,D). In direct contrast, cellular factors implicated in autophagosome early signaling, autophagosome formation and fusion with the lysosome were enriched in the Ub-proteome of ZBR compared to Z-138 cells (Figure 1D and Figure S3B,D). The enrichment of some ALS factors and the reduction of proteasome subunits were confirmed by WB in JBR, another BTZ-resistant cell line, when compared to the parental JeKo-1 cell line (Figure 1E and Figure S3C,E). In summary, our results indicate that in BTZ-resistant cells, the levels of ALS factors associated with the Ub-proteome are increased concomitantly with a reduction in proteasome subunits levels, suggesting a compensatory mechanism that highlights a regulatory crosstalk between ALS and UPS in these cells.

### 2.2. Proteaphagy Is Constitutively Active in BTZ-Resistant MCL Cells

**Figure 2:**
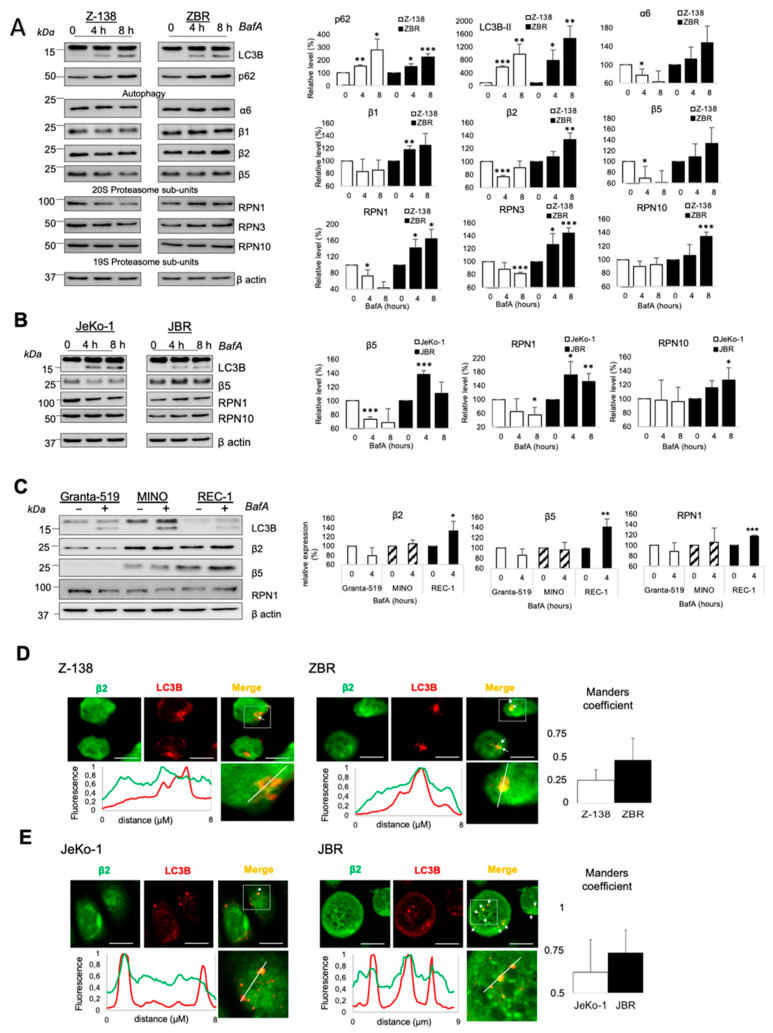
Proteaphagy is activated in BTZ-resistant MCL cells. BTZ-sensitive cells Z-138 (**A**) and JeKo-1 (**B**) and their resistant counterparts, ZBR and JBR, were treated with or without 20 nM BafA for 4 or 8 h. Cells with intrinsic sensitivity (Granta-519) or resistance to BTZ (MINO and REC-1) (**C**) were treated or not treated with 40 nM BafA for 4 h. WB analyses to detect autophagy or 26S proteasome proteins were performed with the indicated antibodies. Quantifications were performed using ImageJ software (*n* ≥ 3; means ± SD; two-tailed Student’s *t*-test, * *p* < 0.05, ** *p* < 0.01, *** *p* < 0.001). Z-138/ZBR (**D)** and JeKo-1/JBR (**E**) were treated with 40 nM BafA for 8 h. Fixed cells were stained with anti-β2 (green) or -LC3B (red) Abs and analyzed by confocal microscopy; scale bar indicates 10 µm. Primary Abs were used: mouse anti-β2 1:100, mouse anti-p62 1:100, rabbit anti-LC3B 1:200, rabbit anti-α2 1:200. Secondary Abs were diluted 1:500 in blocking solution (5% bovine serum albumin, 0.1% Triton ×100 in PBS) and incubated for 30 min at RT. Nuclei were stained with DAPI (P36931, Invitrogen). Looking for co-localization of proteasome and autophagy proteins, normalized values of fluorescence intensity for LC3B (red) and β2 (green) were plotted along the white lines. Co-localization was measured with Manders correlation coefficient calculated in regions of interest with LC3B punctates (*n* ≥ 20; means ± SD; two-tailed Student’s *t*-test, * *p* < 0.05, ** *p* < 0.01, *** *p* < 0.001).

Proteaphagy was proposed to be increased after proteasome inhibition, indicating that this mechanism could be used in physiological conditions to eliminate inactive proteasomes [17,19,28]. We observed that the autophagy inhibitors bafilomycin A (BafA) and chloroquine (CQ) led to the accumulation of autophagy factors (p62 and LC3B) equally well in all cell lines (Figure 2A,B and Figure S4A,B). In sharp contrast, they triggered an increase in the whole-cell levels of 26S proteasome subunits in ZBR and JBR cells and a decrease in Z-138 and JeKo-1 parental (BTZ-sensitive) cells. These results clearly supported the constitutive activation of proteaphagy only in BTZ-resistant cells. To extend these results, we evaluated the accumulation of proteasome subunits in the presence of BafA in several MCL cell lines with distinct inherent resistance/sensitivity to BTZ. We found that the MCL cell line REC-1, with the highest intrinsic resistance to proteasome inhibition [10], accumulated proteasome subunits after BafA treatment. This phenomenon was not statistically significant or not observed in MINO and Granta-519 cell lines showing intermediate and null resistance to BTZ, respectively (Figure 2C and Figure S4C–E). Moreover, the accumulation of proteasome subunits was easier to detect when BTZ treatment was combined with BafA or CQ. Our data suggested that proteaphagy was activated in BTZ-resistant cells. Supporting these observations, immunofluorescence studies revealed a substantial increase in proteasome subunits-containing autophagosomes in ZBR and JBR treated with BafA or CQ plus BTZ when compared to parental Z-138 and JeKo-1 cells (Figure 2D,E and Figure S5A,B). In remarkable contrast with the BTZ-sensitive cell line Z-138, the co-localization of proteasomes with autophagosomes was hitherto observed at basal levels in ZBR cells, and BTZ treatment did not improve this co-localization as much as autophagy inhibitors (Figure S5A). These results indicated that proteaphagy is functionally active in BTZ-resistant cells in the absence of any stimulus and underlined its potential as a hallmark of BTZ-resistance in MCL cells.

### 2.3. p62/Sequestosome-1 Coordinates Proteaphagosome Assembly 

**Figure 3:**
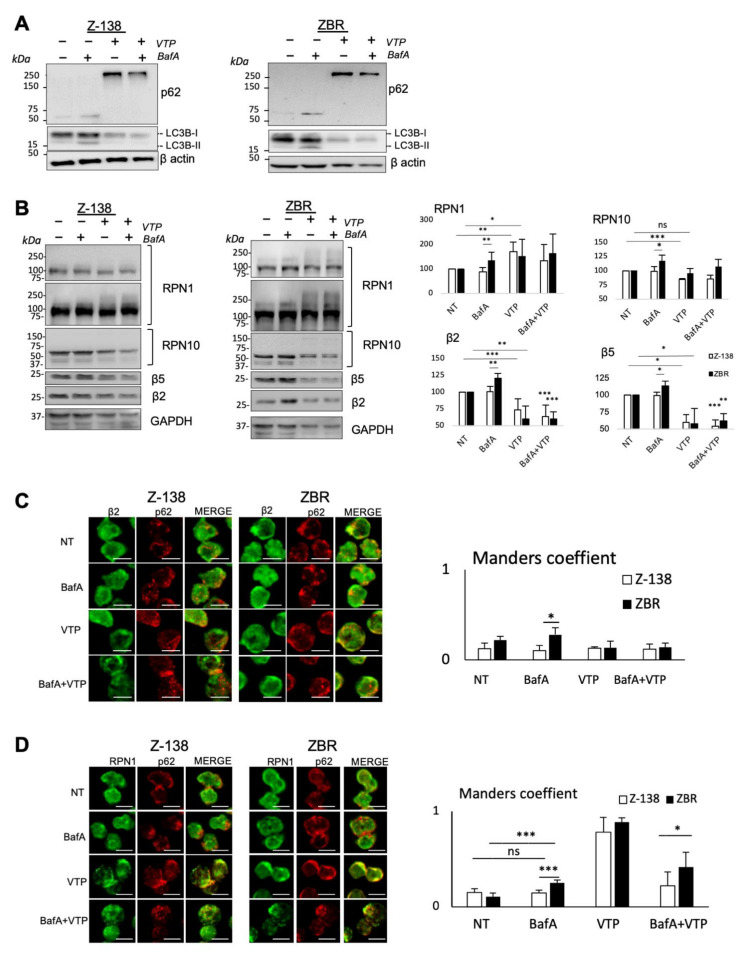
Inhibition of p62 affects the stability and localization of proteasome subunits. Z-138 and ZBR cells were treated with 40 nM BafA, 10 µM VTP or with a combination of BafA/VTP at the same concentrations for 4 or 6 h, respectively. Whole-cell extracts were analyzed by WB to control autophagy flux (**A**) and the total expression of proteasome subunits after treatments (**B**). Relative protein levels were estimated and referred to GAPDH expression by densitometry, using the Image J software. Collected data from three independent experiments were presented in histograms (means ± SD). The two tailed *t*-test unambiguously confirmed the decrease of β2 and β5 subunits in ZBR relative to Z-138. (**C**,**D**) Co-localization of proteasome subunits with autophagosomes after treatment with autophagy inhibitors. Z-138/ZBR were treated or not treated for 4 h with 40 nM BafA or 10 µM VTP or the combined treatment. Fixed cells were stained with anti-β2 antibody (green) and p62 (red) (**C**), or with anti-RPN1 antibody (green) and anti-p62 (red) (**D**). Images were analyzed by confocal microscopy; scale bar indicates 10 µm. Co-localization was measured with Mander’s correlation coefficient calculated in regions of interest containing LC3B or p62 punctates (*n* ≥ 20; mean ± SD; two-tailed Student’s *t*-test, * *p* < 0.05, ** *p* < 0.01, *** *p* < 0.001).

p62 is not only a major player in autophagy but it has also been found to be involved in proteaphagy [19] and in proteasome inhibitor susceptibility in multiple myeloma cells [29]. To explore the role of p62 in proteaphagy, verteporfin (VTP, a clinical-grade small molecule inhibitor of this autophagy receptor) was employed [30,31]. Unlike BafA, VTP inhibits autophagy at an early stage by aggregation of p62 and reduces autophagosome accumulation such that it can be assessed by a reduction of the autophagy marker LC3B [32,33]. Whereas the levels of LC3B and its lipidated form decreased, high-molecular weight aggregates of p62 accumulated after VTP treatment in both Z-138 and ZBR cells. In contrast, BafA treatment accumulated both LC3B forms and p62 monomers. In agreement with the mechanism of action of these drugs when the double treatment was used, VTP stopped the BafA-induced accumulation of LC3B and p62 (Figure 3A). Interestingly, high-molecular weight forms of RPN1 were also observed after VTP treatment. In contrast, β5 and β2 subunits were decreased after VTP treatment in both cell lines (Figure 3B). Accordingly, VTP treatment induced co-localization of p62 with RPN1 but not with β2 subunits (Figure 3C,D). In contrast, BafA accumulated these proteasomal subunits in regions where punctuated autophagosomes were observed, in ZBR but not in Z138 cells (Figure 3B), highlighting a distinct mechanism of action for each inhibitor.

**Figure 4:**
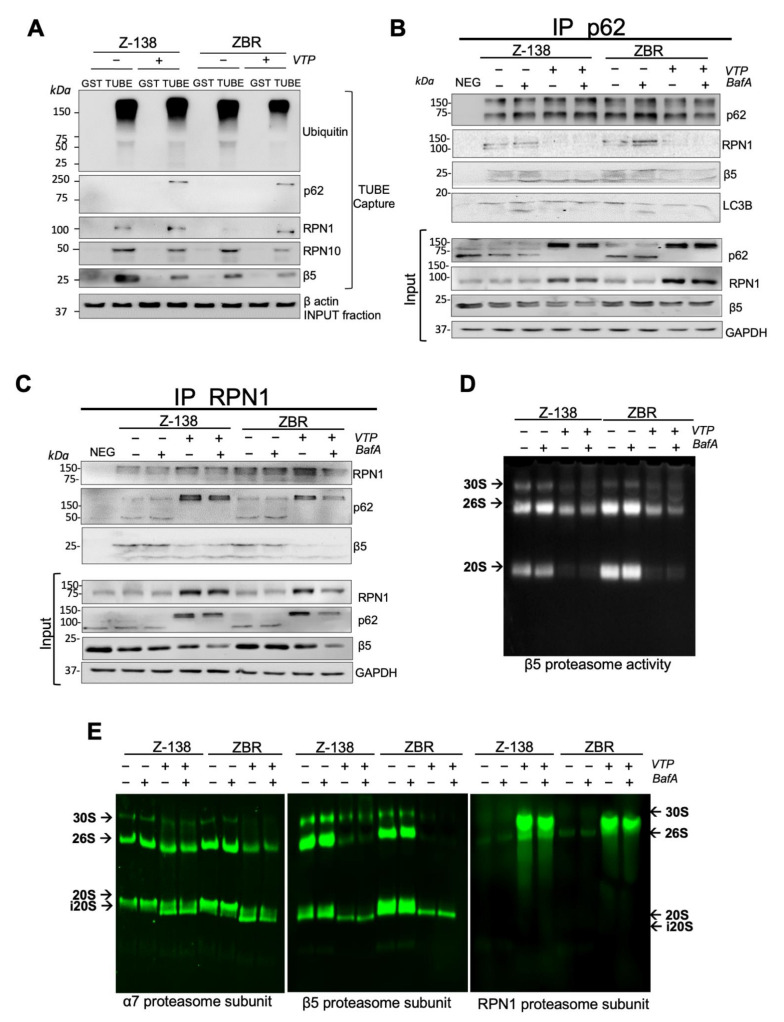
Role of p62 in proteophagosome assembly. (**A**) Ubiquitylated proteins were captured using TUBEs from Z-138 and ZBR treated or not treated with 10 µM VTP for 4 h. GST was used as a control. Indicated fractions were analyzed by WB with the indicated Abs. (**B**,**C**) Cell lysates from Z-138 and ZBR were immunoprecipitated (IP) with anti-p62 and anti-RPN1 Abs. Immunoprecipitated material and input fractions were resolved and subjected to WB with the indicated Abs. (**D**) Cell extracts from Z-138 and ZBR treated or not treated with BafA, VTP or combined treatment were run into non-denaturing native gels. In gel proteasome activity assay was measured using fluorogenic peptides. (**E**) Native complexes separated as in (**D**) were transferred onto PVDF membranes and blotted with the indicated Abs (*n* ≥ 3).

To investigate if high-molecular weight forms of RPN1 could be ubiquitylated, a TUBEs-capture approach was used (Figure 4A). VTP remarkably altered the total ubiquitylation and had a negative impact on the TUBE-capture of RPN10 and β5 proteasome subunits in ZBR cells. Moreover, VTP enhanced the capture of p62 and RPN1 in both ZBR and Z138 cells (Figure 4A). To investigate how VTP affected p62 interactions with distinct proteasome subunits, immunoprecipitation experiments were performed using cell extracts from Z-138 and ZBR cells treated with BafA, VTP or both drugs (Figure 4B). The amount of RPN1 and β5 associated with p62 after BafA treatment was marginally but consistently increased, specifically in ZBR cells. In sharp contrast, the β5 and RPN1 subunits showed a reduced binding to p62 after VTP treatment (Figure 4B). To confirm these observations, RPN1 immunoprecipitation was performed under identical conditions. The level of RPN1 was increased after VTP treatment and this affected the amount of immunoprecipitated RPN1 (Figure 4C). Under these conditions, high-molecular weight forms of p62 were bound to RPN1 while β5 subunits were dissociated (Figure 4C). These results suggest that p62 and RPN1 antibodies recognize distinct complexes differentially affected with BafA or VTP treatments. While the anti-p62 Ab precipitated proteophagosomes in the presence of BafA, the anti-RPN1 Ab precipitated aggregated forms of p62 after VTP treatment. All in all, these results underline the key role of p62 and its association with RPN1 for degradation via proteaphagy. Since VTP treatment could reduce the interaction of RPN1 with the 20S catalytic subunits, this raised the question of its impact on overall proteasome activity. To investigate this point, in gel chymotrypsin-like activity of the proteasome β5 subunit was measured in vitro from VTP-treated MCL cells using reporter peptides [34]. Our results showed that 30S, 26S and 20S activities were reduced after a VTP treatment but unaffected by BafA (Figure 4D). Native gel–WB analysis of α7, β5 and RPN1 subunits revealed that VTP accumulated RPN1 subunits, whereas 20S CPs were reduced and converted into lower-molecular weight forms of the 20S subunit (Figure 4E). According to the in-gel proteasome activity assay, this low-molecular weight form of 20S was not activated (i20S). Overall, our results suggested that p62 contributes to target proteasome subunits in proteaphagic degradation.

### 2.4. Autophagy Inhibition Increases Apoptosis in BTZ-Resistant MCL Cells

**Figure 5:**
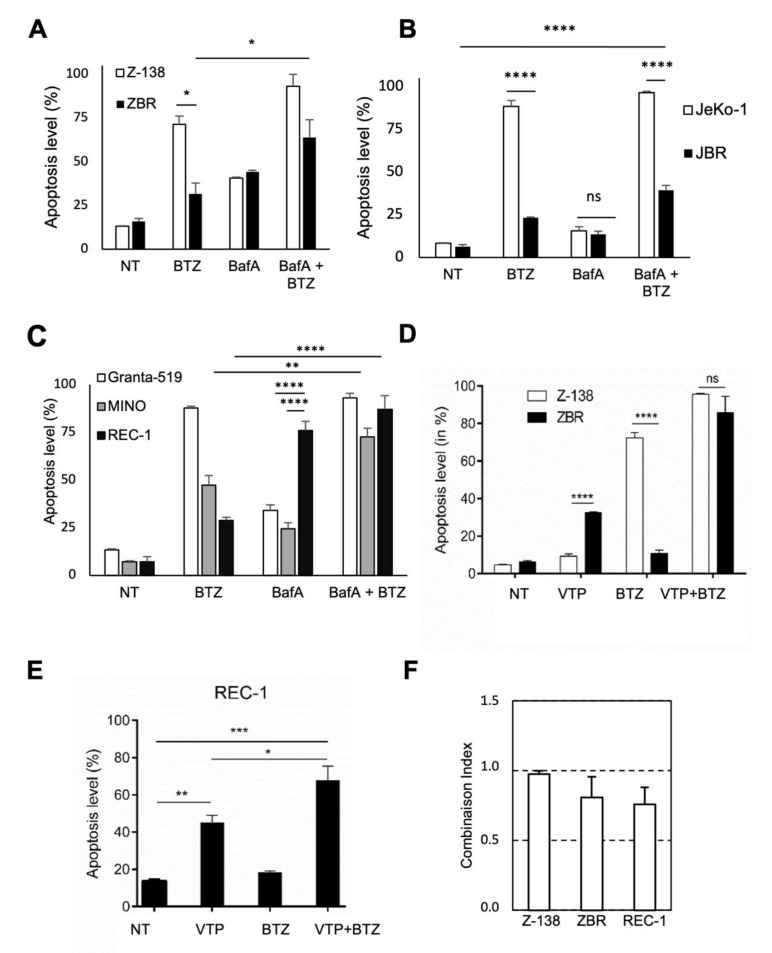
Autophagy inhibition increases apoptosis of BTZ-resistant cells. Flow cytometry analysis of apoptosis in Z-138 and ZBR cells (**A**), JeKo-1 and JBR (**B**) cells or in Granta-519, MINO and REC-1 (**C**) MCL cells treated or not treated for 48 h with 10 nM BTZ, 50 nM BafA or a combination of the drugs, as indicated. Apoptosis levels were measured by annexin V staining in Z-138 and ZBR cells (**D**) treated for 24 h with 5µM VTP, 5 nM BTZ or the combined treatment and in REC-1 cells (**E**) treated for 24 h with 15µM VTP, 5 nM BTZ or the combined treatment (*n* ≥ 3; means ± SD; two-tailed Student’s *t*-test, * *p* < 0.05, ** *p* < 0.01, *** *p* < 0.001, **** *p* < 0.0001). (**F**) From these results, we calculated the combination indexes (CIs) for Z-138, ZBR and REC-1 treated with VTP or BTZ using the Compusyn software (http://www.combosyn.com). The CIs between VTP and BTZ were determined at the constant ratios displayed in (**D**) and (**E**) for Z-138/ZBR and REC-1, respectively. CI values of <1.0 indicate a cooperative effect of the two agents tested in combination.

To explore the impact of autophagy inhibition on the viability of BTZ-resistant cells, pharmacological modulation of apoptosis was evaluated by flow cytometry analysis of ZBR and JBR cells. As expected, compared to their parental cells, both BTZ-resistant cell lines showed a significant reduction in apoptosis levels after a 48h treatment with 10 nM BTZ (Figure 5A,B). However, the 24 h treatment with 20 nM BafA delivered a stronger apoptosis response in ZBR (around 40%) than in JBR cells (around 12%). Both BTZ-resistant and their parental cell lines responded to the BafA treatment to the same extent (Figure 4A and Figure S7). Nevertheless, the combined BTZ plus BafA treatment remarkably stimulated apoptosis in ZBR (up to 70% apoptotic cells) and Z-138 cells (up to 90% apoptotic cells), while this combination exerted relatively modest pro-apoptotic activity (around 40% apoptosis) in JBR cells (Figure 5A,B). Considering the high diversity of MCL genetic backgrounds, we further explored the effects of the same treatments on cell lines that naturally show distinct sensitivity to BTZ (Figure 5C). While BafA alone efficiently killed REC-1 cells (Figure 5C), the BafA–BTZ combination slightly increased the apoptosis rate in these cells when compared with MINO cells (Figure 5C), suggesting that this combination treatment could be more efficient in cells with high bortezomib resistance.

Since p62 plays a critical role in the assembly of proteaphagosomes, we then explored whether its inhibition upon VTP treatment could favorably impact BTZ-mediated apoptosis in resistant cells (Figure 5D–F). Although VTP significantly promoted apoptosis when used alone (plus 30% or 26% cell death in REC-1 and ZBR, respectively), its combination with BTZ achieved a significantly higher degree of cytotoxicity in both ZBR (plus 80% cell death induction, Figure 5D) and REC1 cells (70% cell death induction, Figure 5E), with respective drug combination indexes of 0.81 +/− 0.15 and 0.76 +/− 0.12, indicative of cooperative effects (Figure 5F). Interestingly, this cooperative effect was statistically similar in ZBR and REC-1 cells. 

In order to confirm the participation of p62 in BTZ-mediated cytotoxicity, we used a siRNA approach to knock down the *SQSTM1* (p62-encoding) gene in ZBR cells prior to BTZ treatment. As shown in Figure S6A,B, an 80% reduction was observed in p62 mRNA and protein levels after transfection. This level of expression was maintained upon treatment with standard doses of BTZ. Interestingly, after a 24 h treatment with BTZ, compared to the cells transfected with a non-targeting siRNA, sensitivity to this inhibitor was restored in siSQSTM1-transfected ZBR cells. Following treatment with 7.5 or 10 nM BTZ, the relative cytotoxic activity was recovered by 32% and 38%, respectively (Figure S6C). Accordingly, knocking down *SQSTM1* reduced the mean IC_50_ of BTZ down to 7.33 nM at 24 h, reaching a similar value as the one observed in the parental non-resistant Z-138 cells [10]. These results clearly indicate a role for p62 in the response to BTZ in resistant cells and underline its potential as a target for drug development. 

### 2.5. Inhibition of p62 Reduces the Growth of BTZ-Resistant MCL Tumours In Vivo

**Figure 6:**
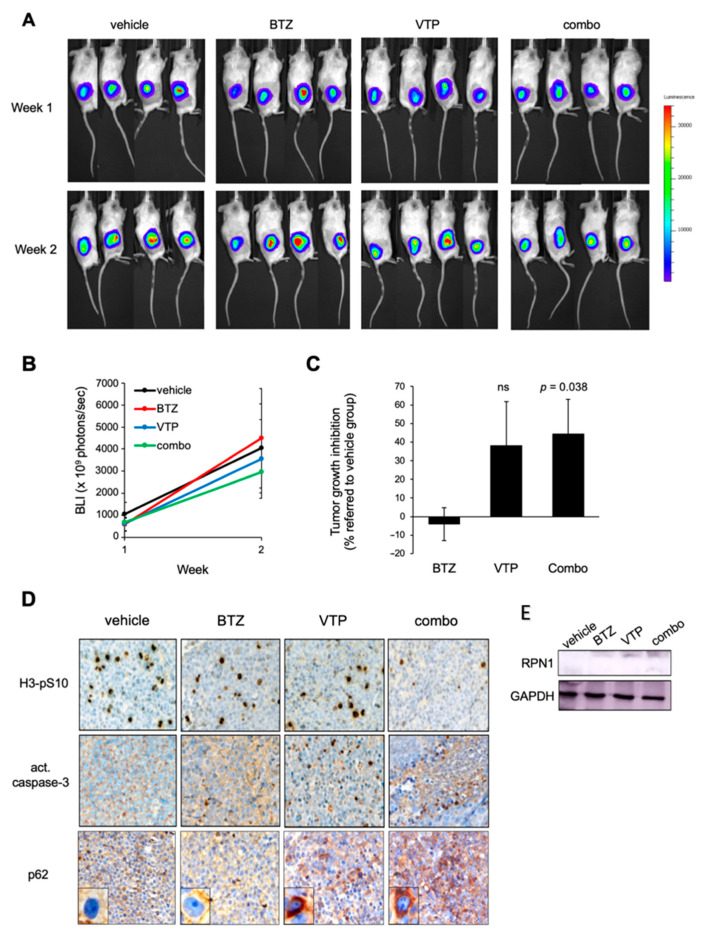
p62 inhibition reduces tumour growth in a BZT-resistant MCL xenograft model. (**A**) NSG mice were subcutaneously injected with REC1-GFP+Luc+ cells and tumor-bearing mice were randomly assigned to one of the following treatment arms (*n* = 4 mice per group): BTZ, 0.5 mg/kg twice weekly (i.p.); VTP, 20 mg/kg twice weekly (i.p.), both agents or equal volume of vehicle, for two weeks. Tumor burden was evaluated at week 1 and week 2 by analysis of the bioluminescence signal. (**B**) Luciferase activity was quantified using Living Image software. (**C**) Tumor volume was evaluated ex vivo using calipers, upon euthanasia of the animals. The inhibition of tumor growth relative to the control group is reported in the histograms. (**D**) Immunohistochemical labelling of phosphorylated histone H3 (H3-pS10) as a marker of proliferating cells and activated (act.) caspase-3 as a marker of apoptosis, and p62 in consecutive tissue sections from four representative tumor specimens (×500, magnification) after 2 weeks of treatment. (**E**) RPN1 protein levels were evaluated by WB analysis in four representative tumor samples after the 2 weeks of treatment. ns, not significant.

To evaluate the potential clinical interest of p62 inhibition in the treatment of BTZ-resistant MCL tumors, REC-1-Luc+ mouse xenografts were treated for two weeks with 0.5 mg/kg BTZ, 20 mg/kg VTP or both drugs. Tumor growth was evaluated for two weeks upon bioluminescent signal and tumor volume was recorded (Figure 6A–D). Consistent with the resistant phenotype of REC1 cells, administration of BTZ as a single agent did not affect tumor burden. VTP-receiving animals underwent a 38% reduction in tumor growth compared to vehicle- or BTZ-treated mice. VTP administration combined with BTZ treatment reduced tumor growth by 44%, reaching statistical significance (Figure 6A,B). Immunohistochemical and WB analysis of tissue sections in representative tumor specimens revealed that single agent VTP was able to reduce the tumor mitotic index and to trigger apoptosis, as shown by lower phospho-histone H3 staining and by the appearance of some nuclei positive for active caspase-3 within the tumors (Figure 6C,D). Most important, VTP-based treatments were associated with a cytosolic accumulation of p62, indicative of inhibited autophagy, together with the consequent accumulation of RPN1. Confirming in vitro results, the VTP–BTZ drug combination was able to improve both p62 and RPN1 accumulation, in association with highest tumor growth inhibition and apoptosis processing (Figure 6C,D). Thus, these in vivo results confirm the therapeutic potential of using autophagy inhibitors to recover the antitumor activity of BTZ in MCL tumors primarily resistant to this agent. 

## 3. Discussion

Bortezomib has been found to significantly improve the prognosis of patients with MM and MCL. However, relapses following this therapy are frequent, while primary resistance to this agent remains a major limitation on the further development of its therapeutic use [5]. Here, using different cellular models of MCL with either acquired or intrinsic resistance, we observed that refractoriness to BTZ is accompanied by ALS activation and, in particular, proteaphagy. Reduction of proteasome subunits in the Ub-proteome is a strong hallmark of BTZ-resistant cells and highlights an important crosstalk between these two major proteolytic pathways in these cells. Other studies have proposed that the reduced expression of 19S proteasome subunits are responsible for the BTZ-resistance found in MM cells [35]. Furthermore, low levels of 19S subunits are at the origin of the resistance to the second-generation PI carfilzomib in MM cells [35,36]. Upon the blockade of autophagy-mediated degradation, both 19S and 20S subunits were accumulated in BTZ-resistant cells with acquired or intrinsic resistance. In intrinsic resistant models, such as REC-1 cells, the accumulation of proteasome subunits upon autophagy inhibition was easier to highlight. The reduction in proteasome subunits within the Ub-proteome was also observed in JBR cells. JBR and its parental cell line JeKo-1 carry mutations/deletions of relevant genes, such as *TP53*, *MLL2*, *DCP1B* and *TRMP6* [37]. Despite these genetic alterations, proteaphagy was observed in JBR, indicating that active proteaphagy is a well-preserved mechanism present in cells that resist BTZ treatment independently of the genetic background of the tumor cells. 

Our co-localization analyses employing two distinct markers of autophagy (LC3B and p62), clearly indicate that 20S proteasome subunits are accumulated in autophagosomes after treatment with autophagy inhibitors, indicating ongoing proteaphagy. Cells may employ this mechanism in the presence of deficient proteasomes to restore proteostasis [15,16]. Interestingly, the use of distinct autophagy inhibitors (CQ, BafA or VTP) did not lead to the same results in ZBR and JBR in terms of timing and efficiency in accumulating proteasome subunits nor in the capacity to drive apoptosis alone or in combination with BTZ. This suggests that the molecular mechanisms engaged by these drugs are not exactly the same [31,38]. Blocking autophagy at the level of the p62 receptor appears to negatively affect the assembly of the 26S proteasome and results in a dissociation of the 20S from the 19S subunits. Since VTP could trigger oxidative stress in a light-dependent manner [30] and produce reactive oxygen species (ROS), such as singlet oxygen and radical species [39], ROS contribute to the observed effects on proteasome stability and activity [40]. In any case, RPN1 is likely fulfilling a relevant role in the response to VTP since, in contrast to other proteasome subunits tested, its integration in the ubiquitin proteome is increased after treatment with this drug. 

The role of p62 in the response to BTZ in multiple myeloma has been previously suggested [29]. Although the mechanism of BTZ-resistance acquisition is not fully clear, it appears that it could be common to other proteasome inhibitors, such as carfilzomib, since p62 is directly or indirectly implicated in this process [41]. Indeed, knocking down SQSTM1 in ZBR cells significantly potentiates BTZ cytotoxicity (Figure 6), supporting the role of p62 in BTZ resistance in MCL models. Actual options for chemical inhibitors of p62 are limited. The XRK3F2 inhibitor that specifically targets the ZZ domain of p62 [42] can overcome BTZ resistance in MM, independently of *TP53* status [43,44]. We therefore tested the p62 inhibitor XRK3F2 in BTZ-resistant MCL cells, but its cytotoxic effects were less pronounced than the ones observed with VTP (data not shown). This suggest that, in addition to p62, other factors might play a crucial role in the response/resistance to proteasome inhibitors. 

Late-stage autophagy inhibitors also induced distinct apoptosis levels in BTZ-resistant MCL cells. In inherent BTZ-resistant cell models, the resistance to BTZ seems to be positively correlated with their dependency on autophagy, and BafA alone compromises cell viability (e.g., results from REC-1). However, the combined treatment BTZ/BafA could be more efficient to kill cells with intermediate levels of resistance (e.g., results from MINO) [37]. Interestingly, the most efficient single treatment was VTP and the combination index indicated that cooperative effects can be observed when combined with BTZ. These observations were also validated in xenografted mouse models using the REC-1 cell line. 

## 4. Materials and Methods 

### 4.1. Antibodies

Antibodies (Abs) anti-P4D1 ubiquitin, -β2, -PSMD2, -p62 were purchased from Santa Cruz Biotechnology (CA). Abs anti-α6 and -PSMD3 were obtained from Invitrogen. Anti-β-actin Ab was from Abgent. Anti-annexin 5 Ab was from BD Science. Abs anti-β1, -RPN10 and -RPT4 were from Enzo Life Science. Anti-β5, -PSMA2, -LC3B, -ADRM1, -AMBRA1 and -HYOU1 were from Cell Signaling Technology (Danvers, MA, USA). Abs anti-mTOR and -S100A9 were from Sigma (St. Louis, MO, USA). Abs anti-UVRAG, -TOLLIP, -VPS33A, and -VTI1A were from Thermo Fisher Scientific (Waltham, MA, USA). Donkey anti-rabbit TexRed and donkey anti-mouse FITC secondary Abs were from Jackson Immunoresearch. Goat anti-mouse Star 635 was from Abberior and Donkey anti-Rabbit Alexa Fluor 594 was from Invitrogen. Fluorescent secondary Abs IRDye^®^ 800CW and IRDye^®^ 680RD were from LICOR. Peroxidase Goat Anti-Rabbit IgG and Peroxidase Rabbit Anti-Mouse IgG were from Jackson Immunoresearch. 

### 4.2. Reagents

Ac-nLPnLD-amc, Boc-LSTR-amc and Suc-LLVY-amc peptides were purchased from Bachem AG (Bubendorf, CHE). Marizomib, chloroquine and verteporfin were from Sigma (St. Louis, MO), bortezomib was from Tebu bio (Le Perray en Yvelines, France), bafilomycin A1 and everolimus were from Invivogen (San Diego, CA, USA).

### 4.3. Cell Culture

ZBR and JBR were derived from Z-138 and JeKo-1, respectively [10]. Briefly, JeKo-1 and Z-138 cells were initially treated for 96 h with 10 nM BTZ and then cultured in drug-free media containing 20% fetal calf serum. After cell growth recovered, cells were treated with BTZ for an additional 72 h, and the selection cycle was repeated at the same concentration of BTZ until cell growth recoveries were obtained within two weeks. At this point drug concentration was increased to the next step (12 nM, 15 nM, 20 nM and, finally, 30 nM). After repeated rounds of selection with the 30 nM dose over a period of 5 months, the resistant cell lines were established and designated JBR and ZBR. All cells were cultured in RPMI 1640 with 2 mM L-glutamine, 100 Units/mL penicillin, 100 µg/mL streptomycin and 10% foetal bovine serum (FCS), excepting JeKo-1 and JBR (20% FCS). Cells were incubated at 37 °C, 5% CO_2_. Cell line authentication was based on short tandem repeat (STR) profiling by DSMZ services (Braunschweig, Germany).

### 4.4. TUBEs Capture

The capture of ubiquitylated proteins for mass spectrometry analysis was performed as described in [25]. For all other TUBE-captures, 10^7^ cells were treated and used according to the protocol reported in [21,22].

### 4.5. Mass Spectrometry Analysis

TUBEs–mass spectrometry analysis was performed as reported [27,45]. Mass spectrometry data are available in the PRIDE database: http://www.ebi.ac.uk/pride accession number “PXD011867”. Last access: 10 February 2022.

A stringent protein identification filter was selected, retaining only proteins with a false discovery rate (FDR) below <1%. Spectral counts were summed up for each matching peptide for a given protein and spectral counts obtained in GST control samples were subtracted using the statistical programming language R. Negative and zero values were set to one to enable log_2_ transformation of the quantitative data. The data were then log_2_ transformed and quantile normalized across samples. Proteins with a more than two-fold difference regulated between Z-138 and ZBR that could be reproduced in all three replicas were maintained for further western blot validation and functional evaluation. From the 263 selected proteins with more than two-fold difference between Z-138 and ZBR that could be reproduced in all three replicas, 60 were UPS and ALS factors. Among the 263, 123 were more abundant in ZBR and 140 less abundant in ZBR. These proteins were maintained for further dry and wet analyses. 

### 4.6. IPA Analysis 

Analysis using ingenuity pathway analysis (IPA^®^, QIAGEN Redwood City, CA USA, www.qiagen.com/ingenuity, accessed on 1 January 2017) integrated canonical signaling pathways associated with Z-138 and ZBR cells (*p*-value < 0.05, calculated by Fishers’s exact test). The calculated *p*-values determine the probability that the association between proteins in the dataset and the canonical pathways is explained by chance alone. 

### 4.7. Database Search

The obtained data from the 120 LC–MS runs were searched using VEMS [46] and MaxQuant [47] using both higher-energy induced collisional dissociation (HCD) and ETD scoring. A standard human proteome database from UniProt (3AUP000005640) including common contaminating proteins was used [48]. Permuted protein sequences, where Arg and Lys were not permuted, were included in the database. The total number of protein entries in the database was 140,149. Trypsin cleavage allowing a maximum of 4 missed cleavages was used. Carbamidomethyl cysteine was included as fixed modification. Methionine oxidation (UNIMOD: 35), N-terminal protein acetylation (UNIMOD: 1), GG tag (UNIMOD: 121) on lysine and LRGG tag (UNIMOD: 535) on lysine deamidation (UNIMOD: 7) of asparagine and glutamine were included as variable modifications. Five ppm mass accuracy was specified for precursor ions and 0.5 m/z for fragment ions. The FDR for protein identification was set to 1% for peptide and protein identifications. No restriction was applied for minimal peptide length for the VEMS search. Identified proteins were divided into evidence groups [49].

### 4.8. Bioinformatics Analysis

Gene ontology analysis (http://geneontology.org) was made using the R packages GO.db [50] and KEGG.db [51] for the proteins associated with the functional categories in the heatmaps. Venn diagrams were made with the R package VennDiagram [52].

### 4.9. Western Blotting

Western blot analysis was performed as previously reported [21,22]. Pictures were acquired using West Femto ECL (34096 Thermo Fisher Scientific, Waltham, MA, USA) with PXI4 (Syngene, Bangalore, India) and GeneTools (Syngene, Bangalore, India). Quantifications were performed using ImageJ software. The density of each protein of interest was then normalized against the density of the control housekeeping protein (β-actin or GAPDH). Relative protein levels under treatment conditions were calculated with respect to the control basal condition. 

### 4.10. Immunoprecipitation

Abs (3–5 μg per point) were incubated 2 h at 4 °C with 30–40 μL of protein A magnetic beads (Millipore). Abs were then crosslinked as reported [25]. Cell pellets were lysed with 500 µL TUBE lysis buffer [21]. After 5 min incubation on ice, samples were centrifuged for 30 min at 13,000 rpm at 4 °C. Supernatants were incubated for 1 h at 4 °C with 30 µL of DMP-crosslinked beads-Abs. After 5 washes with PBST 0.05%, pulled down proteins were eluted in 100 µL 1.5X boiling buffer (4% SDS, 20% glycerol, 120 mM Tris-HCl pH 6.8) before analysis by immunoblotting.

### 4.11. Immunofluorescence Microscopy

Immunofluorescence analysis of Z-138/ZBR and JeKo-1/JBR was performed with the same reported protocol [10]. Abs were incubated overnight at RT in blocking solution (5% bovine serum albumin, 0.1% triton ×100 in PBS). Images from Figure 2 and Figure S5 were acquired with a Zeiss Observer Z.1 Microscope implemented with the Zeiss LSM 510, C-apochromat objective 40×/1.20 W (UV-V15-IR, Torr), using the Zen 2009 software package (Zeiss, Jena, Germany). Images from Figure S5 were acquired with a Nikon confocal C1-Si with 40× oil immersion objective 1.3 NA, using NIS-Elements Software (Nikon, Minato-ku, Tokyo). Pictures were assembled with Adobe Photoshop 7.0 (Adobe, San Jose, CA, USA). Images were not modified except for adjustments of levels, brightness and magnification. Co-localisation analyses and Mander’s co-localisation coefficient calculations were performed using ImageJ. 

### 4.12. Native Gel Electrophoresis and In Gel Proteasomal Activity Assay

The analysis of proteasome complexes and activity were performed by native gel electrophoresis, as reported [34,53]. Ten million fresh or rapidly thawed cells were used for each experimental point. Thirty micrograms of total protein were migrated per well in NuPAGE™ Novex™ 3–8% Tris-Acetate Protein Gel (Thermo Fisher Scientific, Walthan, MA, USA). Migrations were performed in native gel electrophoresis buffer (NG buffer) (90 mM Tris-borate, 0.1 mM EDTA, 5 mM MgCl_2_, 0.5 mM ATP, 0.5 mM DTT) at 150 volts for 3 h. The intrinsic activity of native proteasomes was analysed in gel by 20 min incubation in NG buffer supplemented with 100 µM Suc-LLVY-AMC (Bachem, Bubendorf, Switzerland) at 37 °C. The amount of cleaved AMC fragment was imaged with Syngene NuGenius. Native gels were then washed twice for 10 min in 10× Tris-glycine–SDS Laemmli buffer (0.25 M Tris, 1.92 M glycine, 1% SDS, pH 8.6), followed by a final wash in 1× Tris-glycine–SDS Laemmli buffer. Gels were transferred in PVDF membranes (0.45 µm pore size, Immobilon-P, Merck, Kenilworth, NJ USA) overnight at 40 volts at 4 °C. Membranes were blotted and analysed for the proteins of interest.

### 4.13. Flow Cytometry

Cells seeded at 4 × 10^5^/mL in 12- or 24-well plates were collected and pelleted by centrifugation at 125× *g* for 5 min. Pellets were resuspended and cells were stained for 20 min with 100 μL 1× Annexin V Buffer (BD Pharmingen, San Jose, CA, USA) with 1:100 FITC-Annexin V (BD Pharmingen). Then, 300 μL of Annexin Buffer were added to the samples and 10^4^ events were collected for each experiment. Means ± SD of all events were calculated from the population.

### 4.14. In Vivo Experiments

REC1-GFP-Luc cells were produced and inoculated subcutaneously in 7-week-old NSG mice, as previously described [54]. Animals were randomly assigned to 4 equivalent cohorts and treated intraperitoneally with 0.5 mg/kg BTZ and/or 20 mg/kg VTP, twice weekly for two weeks. Animals in the control group were dosed with an equal volume of vehicle. Tumour engraftment was determined weekly following injections of the mice with 75 mg/kg D-luciferin (AnaSpec, Fremont, CA USA) and bioluminescence recording with a Xenogen IVIS Spectrum (Perkin Elmer, Waltham, MA, USA). After two weeks, the animals were then sacrificed and tumours were measured ex vivo by external calipers. Tumour samples were formalin-fixed and paraffin-embedded and subjected to immunohistochemical analysis following a previously described procedure [11]. The primary Abs used were phospho-histone H3 and cleaved-caspase-3 (Cell Signaling Technology, Danvers, MA, USA) and p62 (Tebu-Bio, Le Perray en Yvelines, France). Images were acquired using an Eclipse microscope with the NIS-Elements Viewer software (Nikon Minato-ku, Tokyo). One representative tumour of each group was used for the quantification of RPN1 protein levels by SDS-PAGE, as previously published [55], using an anti-RPN1 monoclonal antibody (Tebu-Bio). Animal handling was performed following protocols approved by the Animal Ethics Committee of the Autonomous University of Barcelona (protocol #37/18).

### 4.15. Quantification and Statistical Analysis 

All experiments were repeated at least 3 times unless stated in the figure legend. Two-tailed unpaired Student’s *t*-tests were applied for comparisons between two groups. The data are presented as the means ± SD except where stated otherwise. *p*-values < 0.05 were considered statistically significant.

## 5. Conclusions

Our results indicate that manipulating proteaphagy could be used as a strategy to treat BTZ-resistant MCL cells since they become “addicted” to autophagy and therefore hypersensitive to the inhibition of both proteolytic pathways. Since proteaphagy is permanently activated in BTZ-resistant cells but absent from untreated BTZ-sensitive cells, this process might only concern inactive proteasomes under these conditions. However, since BTZ is still blocking some proteasome activity in BTZ-resistant cells, further investigations are required to elucidate whether proteaphagy distinguishes inactive from active proteasomes. Since proteaphagy eliminates the target of BTZ (20S proteasomes), the sensitivity to this treatment is reduced in BTZ-resistant cells. We propose proteaphagy as a new mechanism contributing to the lack of sensitivity to BTZ observed in BTZ-resistant cells. The concomitance of proteaphagy with other compensatory processes previously shown to impair BTZ activity in MCL, such as the activation of the unfolded protein response [10] or the plasmacytic differentiation program [11], cannot be ruled out.

A new generation of PIs have been subsequently developed to reduce toxicity and overcome BTZ-resistance [56]. Marizomib constitutes a promising example that acts on the three catalytic subunits of the proteasome and improves apoptosis when combined with distinct autophagy inhibitors on BTZ-resistant cells [7,56]. Although our study has some limitations, such as the use of a single disease model and the absence of a co-culture experiment associating primary MCL cells with stromal layer cells mimicking the physiological environment of MCL, we are confident that our approach will be robust enough to allow the identification of the multifactorial mechanisms that mediate inherent and acquired resistance to these second generation PIs and fulfill the final objective of enhancing the therapeutic efficacy of these agents in clinics.

## Data Availability

Mass spectrometry data are available in the PRIDE database: http://www.ebi.ac.uk/pride accession number “PXD011867”. Last access: 10 February 2022.

## Supplementary Materials

The following supporting information can be downloaded at: https://www.mdpi.com/article/10.3390/cancers14040923/s1, Supplementary materials contain supplementary methods and supplementary figures and legends as follows: Figure S1: TUBEs–MS procedure to identify the ubiquitin proteome from MCL cells, Figure S2: Heat map representation of the 263 proteins enriched or reduced in studied MCL cell lines, Figure S3: Analysis of the UPS and ALS proteins identified in our TUBEs–MS analysis, Figure S4: Inhibition of autophagy revealed an active proteaphagy in distinct BZT-resistant MCL cell lines, Figure S5: Co-localization of proteasome subunits with autophagosomes in distinct MCL cell lines, Figure S6: Silencing of p62 improves sensitivity to BTZ in BZT-resistant MCL cells, Supplementary Table S1: Excel file with the following MS data can be obtained at: the PRIDE database: http://www.ebi.ac.uk/pride accession number “PXD011867”. Last access: 10 February 2022.
