# Supplementary material for: Constitutive Activation of p62/Sequestosome-1-Mediated Proteaphagy Regulates Proteolysis and Impairs Cell Death in Bortezomib-Resistant Mantle Cell Lymphoma
Source: Cancers (Basel). 2022 Feb 12;14(4):923. doi: 10.3390/cancers14040923 (PMC8869867; doi:10.3390/cancers14040923)
Supplement: Supplementary file 1 [file cancers-14-00923-s001.zip › Supplementary materials Quinet et al.pdf]

Supplementary materials

Constitutive activation of p62/Sequestosome-1-mediated proteaphagy regulates proteolysis and impairs cell death in bortezomib-resistant mantle cell lymphoma

Grégoire Quinet1,a, Wendy Xolalpa2,a, Diana Reyes-Garau3, Núria Profitós-Pelejà3, Mikel Azkargorta4, Laurie Ceccato1, Maria Gonzalez-Santamarta1, Maria Marsal5, Jordi Andilla5, Fabienne Aillet2, Francesc Bosch6, Felix Elortza4, Pablo Loza-Alvarez5, Brigitte Sola7, Olivier Coux8, Rune Matthiesen9, *, Gaël Roué3, *, Manuel S. Rodriguez1, *

Supplementary Methods

RNA interference assay

For transient down-regulation assays, ZBR cells (7 × 106) were electroporated with a Nucleofector Device (Lonza, Verviers, Belgium) using the C-005 program. Briefly, cells were resuspended in 100 µl of Ingenio Electroporation Solution (Mirus, Madison, WI, USA) containing either 2.5 µM of a mix of 3 different small-interfering RNA (siRNA) sequences from TriFECTa Kit DsiRNA Duplex (Integrated DNA Technologies, Coralville, IA, USA) targeting the SQSTM1 gene or a non-silencing (scramble) siRNA. After transfection, cells were transferred to culture plates and the different concentrations of bortezomib were added 8 h later. Proliferation was checked 24 h later by a Cell Titer-Glo Luminiscent Cell Viability Assay (Promega, Madison, WI, USA), performed as per manufacturer's instructions. The IC50 was defined as the concentration required to reduce proliferation by 50% and was calculated with the GraphPad Prism 8 (GraphPad Software).

RNA extraction and Quantitative Real-Time PCR.

Total RNA was extracted with TRIzol Reagent (Thermo Fisher) and retrotranscribed with the High-Capacity cDNA Reverse Transcription Kit (Applied Biosystems). mRNA levels were quantified using quantitative real-time PCR (qRT-PCR) with the GoTaq® qPCR Master Sybr Green Mix (Promega, Madison, USA). Relative expression was analyzed using the QuantStudio5 Real-Time PCR systems (Thermo Fisher) with the ΔΔCT method and normalized with a housekeeping gene (GAPDH, B-ACTIN and or B2M).

Supplementary tables

Supplementary Table 1. Excel file with the following MS data: 1.1 All proteins (-GST spect.counts); 1.2 Selected proteins repl=3 fold=2; 1.3 Ub Signatures (GG and LRGG).

Supplementary Figures and legends

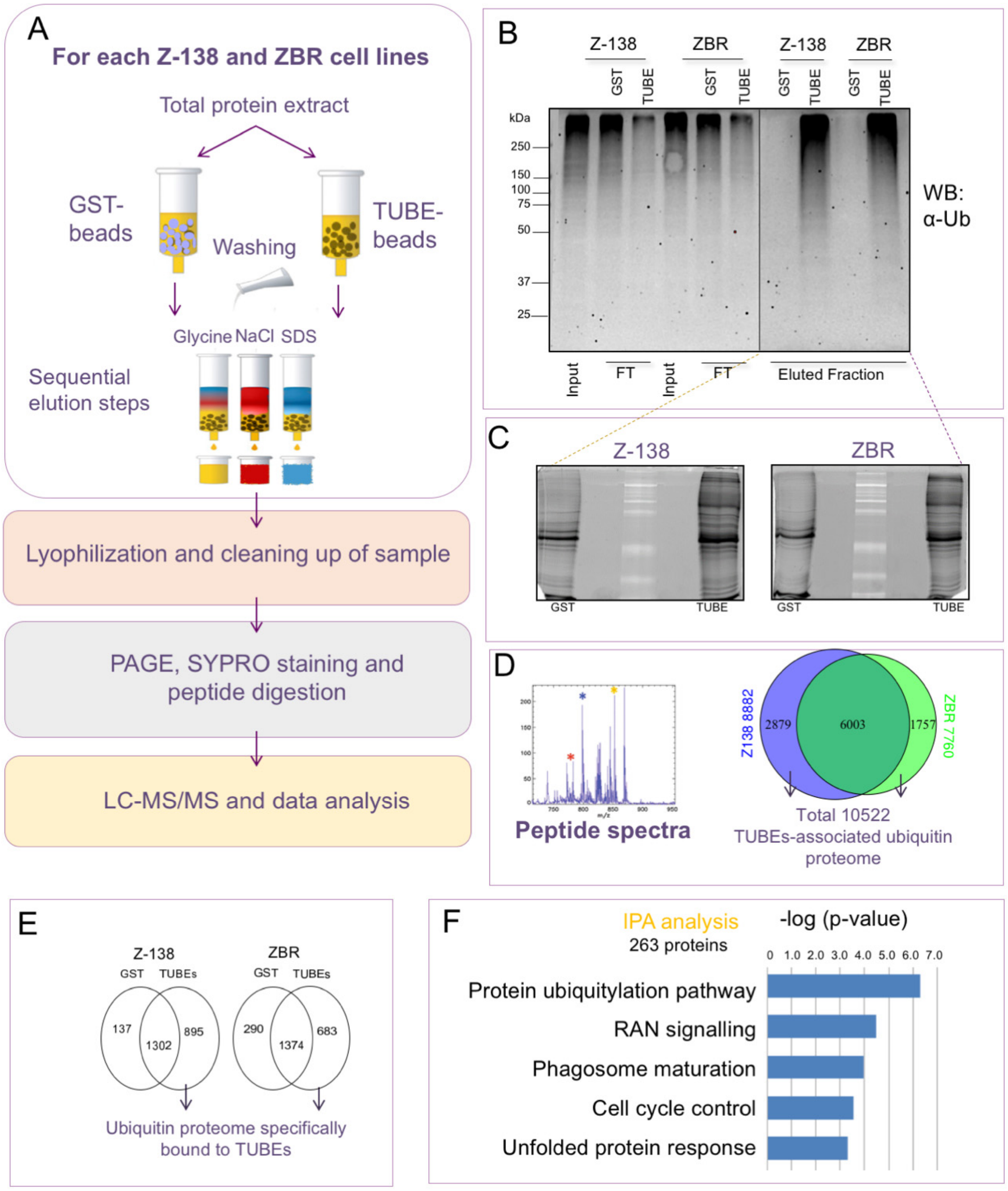

Figure S1. TUBEs-MS procedure to identify the ubiquitin proteome from MCL cells. (A) Full scheme of the procedure followed to identify the TUBE-associated ubiquitin proteome. (B) Input, Flow-through (FT) and eluted fractions were analysed by WB with anti-ubiquitin antibody. (C) GST control and TUBE-eluted fractions were Sypro Ruby stained and sliced gel digested with trypsin. (D) Identified peptide-spectra found in distinct cell lines were compared. (E) The ubiquitin proteome specifically bound to TUBEs was retained for further analysis. In this way 895 proteins and 683 proteins from Z138 or ZBR respectively were submitted to additional enrichment filtering (see methods). The selected 263 proteins were analyzed by ingenuity (IPA) and the top 5 more affected functions were uncovered.

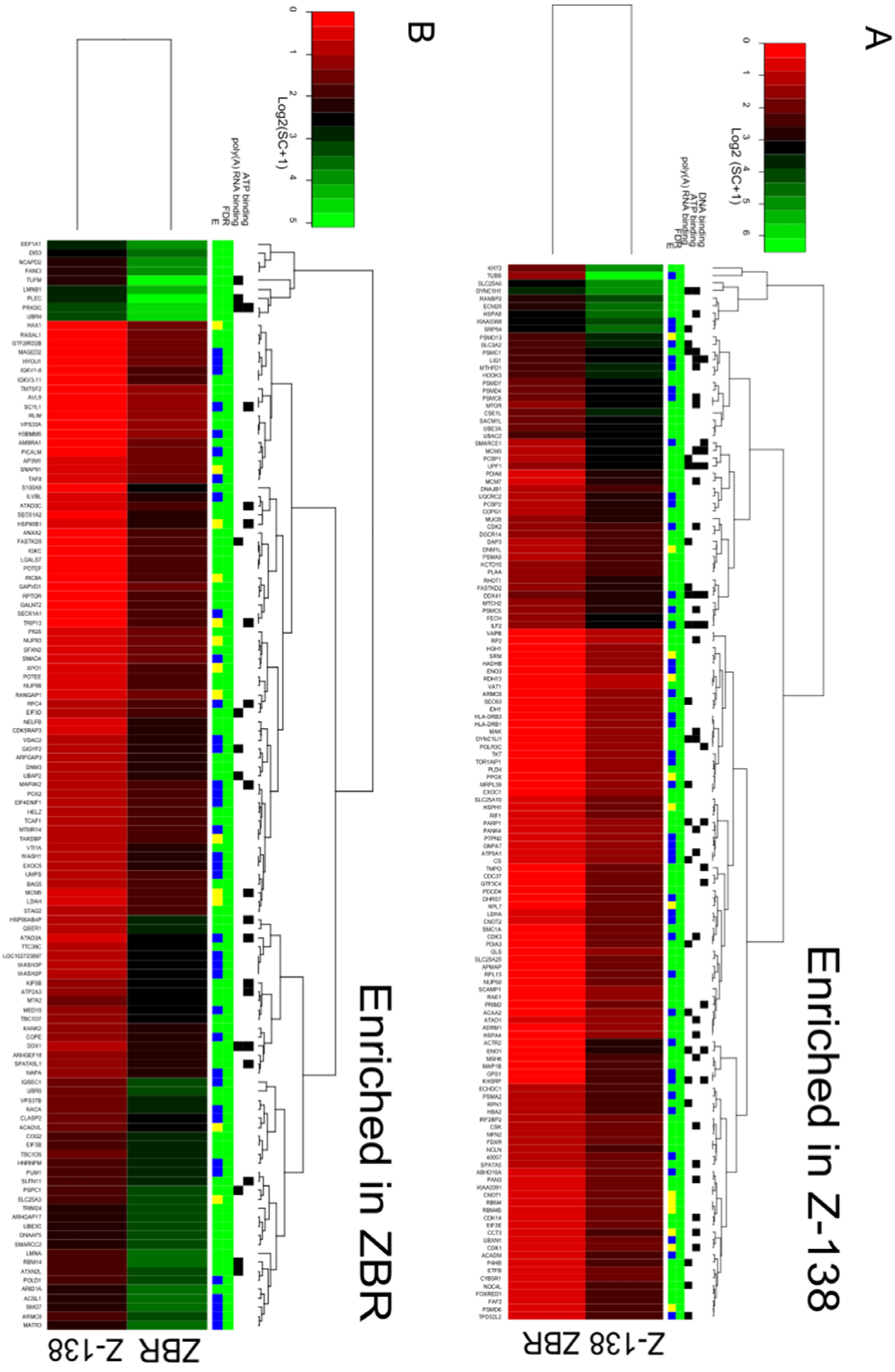

Figure S2. Heat map representation of the GST subtracted log2 spectral counts for 263 proteins enriched or reduced in studied MCL cell lines (averaged over three replications). (A) Proteins enriched in the parental BTZ-sensitive cell line Z138. (B) Proteins enriched in the BTZ-resistant cell line ZBR. Upper panel: E indicates the protein evidence level (Matthiesen R et al 2012) were green indicates evidence level 1 (identified by unique peptides), blue level 2 (identified by semi-unique peptides) and yellow level 3 (identified by ambiguous peptides). FDR indicates the false discovery rate at the level of the protein score which for all cases equaled 0. Black boxes indicate functional annotation of the proteins.

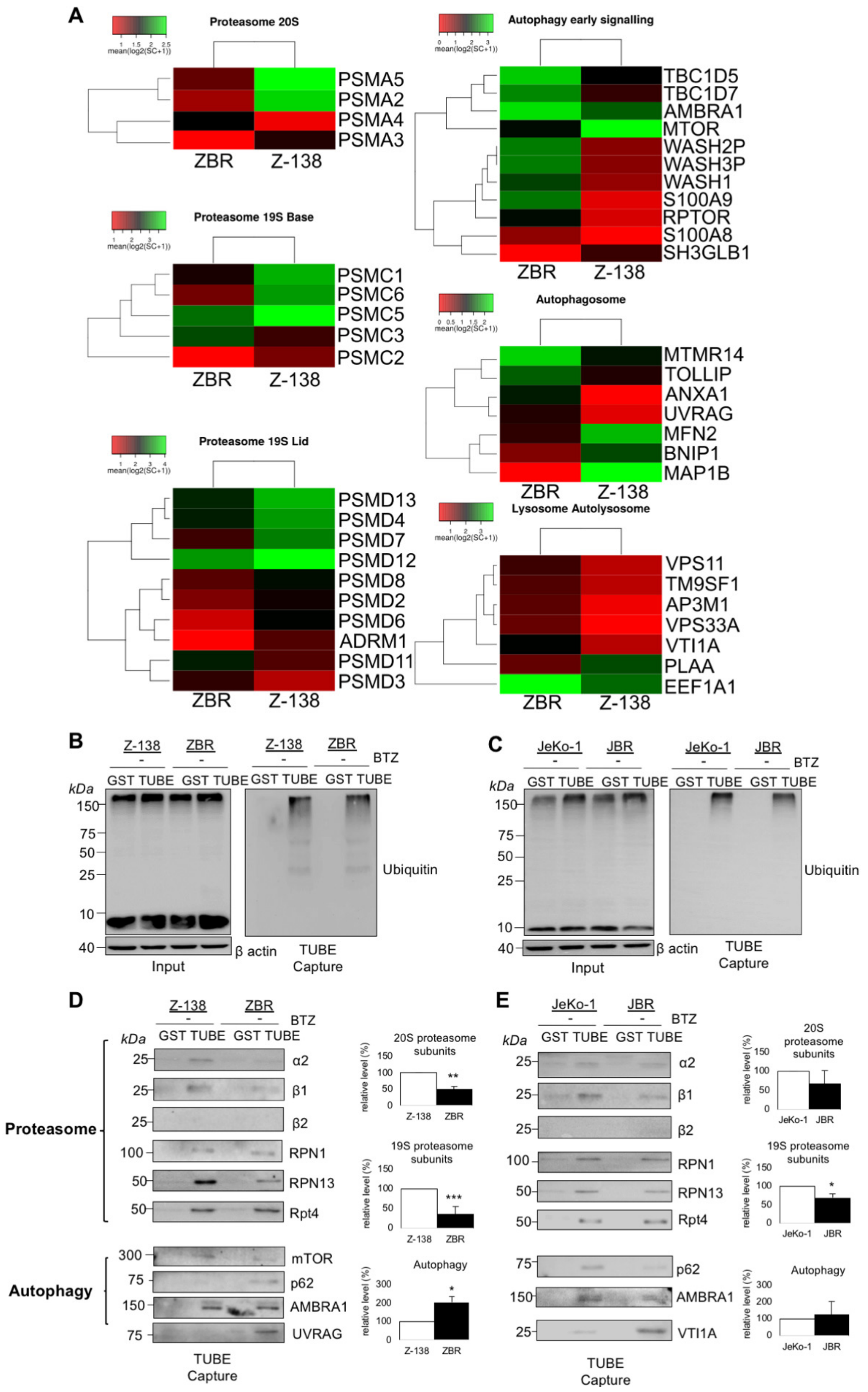

Figure S3. Analysis of the UPS and ALS proteins identified in our TUBEs-MS analysis. (A) Heat map representation of the identified proteins from UPS and ALS. Proteins enriched or reduced in ZBR cells compared

to the parental Z-138 cell line are represented. Red = reduction; Black= no enrichment or reduction; Green = enrichment. According to the color scale, low or high changes are illustrated. (B and C) Ubiquitylation pattern in Z-138 vs ZBR and JeKo-1 vs JBR cells, respectively. Cells were not treated with BZT in this analysis. (D and E) Specific cellular factors captured by TUBEs, under the same conditions as B and C. GST was used as control. Input and captured fractions were analysed by WB with the indicated antibodies. The densities of proteins calculated for the 19S, 20S and autophagy factors are the mean of all single values. Quantifications were performed using ImageJ software ($n \geq 3$; mean ± SD; two-tailed Student's t-test, *$p<0.05$, **$p<0.01$, p***<0.001).

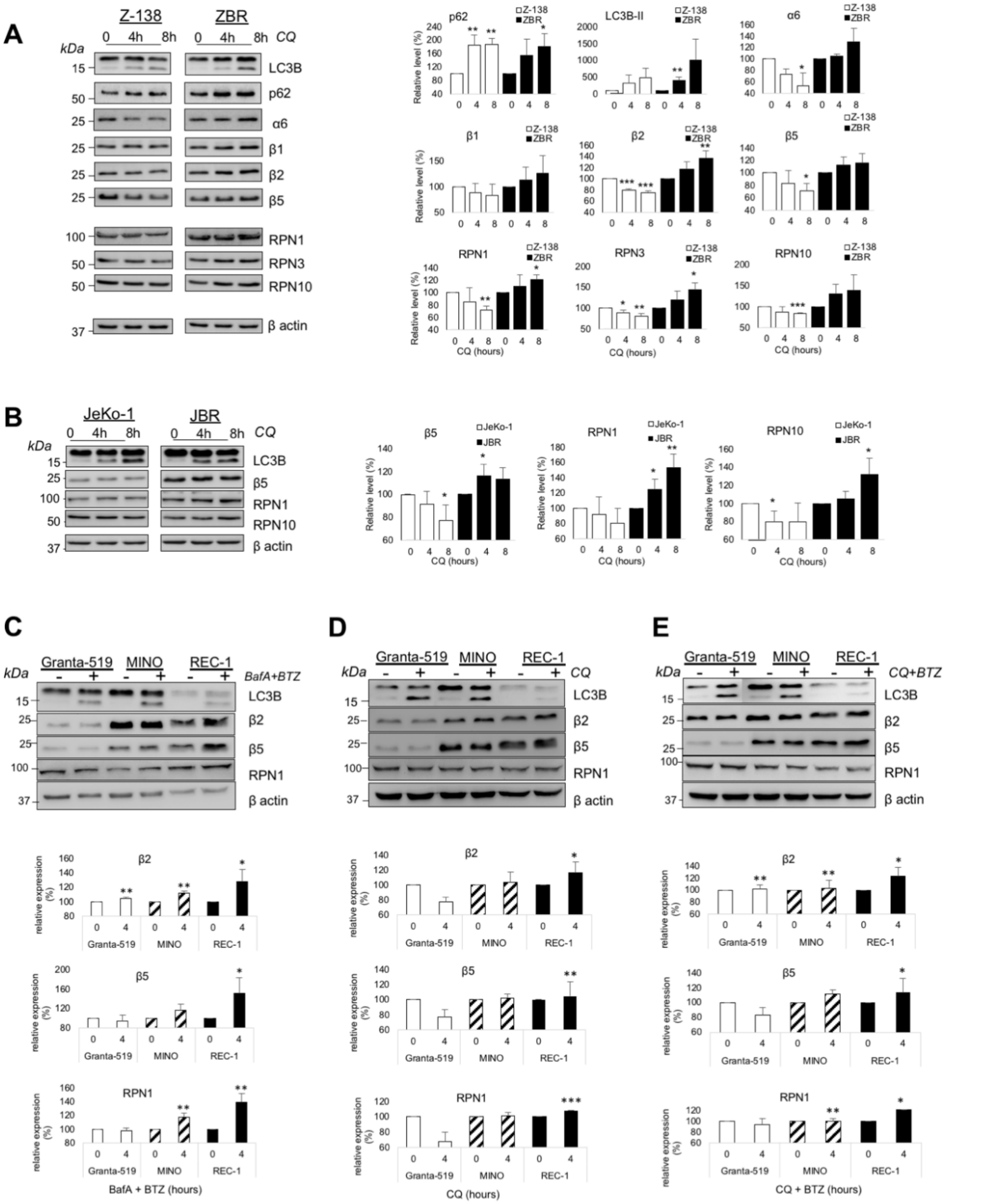

Figure S4. Inhibition of autophagy revealed an active proteaphagy in distinct BZT-resistant MCL cell lines. (A) BTZ-sensitive cells Z138 their resistant counterpart ZBR were treated or not during 4 or 8 hours with 40μM CQ. (B) BTZ-sensitive cells JeKo-1 their resistant counterpart JBR were treated or not during 4 or 8 hours with 40μM CQ. MCL cells Granta-519, MINO and REC-1 were treated during 4 hours with the combined treatment BafA 40nM and BTZ 10nm (C), with CQ 40 μM (D) or with the combined treatment CQ 40 μM and BTZ 10nm (E). WB analyses were done with the indicated antibodies, and detected proteins were quantified by ImageJ. (n ≥ 3; mean ± SD; two-tailed Student's t-test, *p<0.05, **p<0.01, p***<0.001).

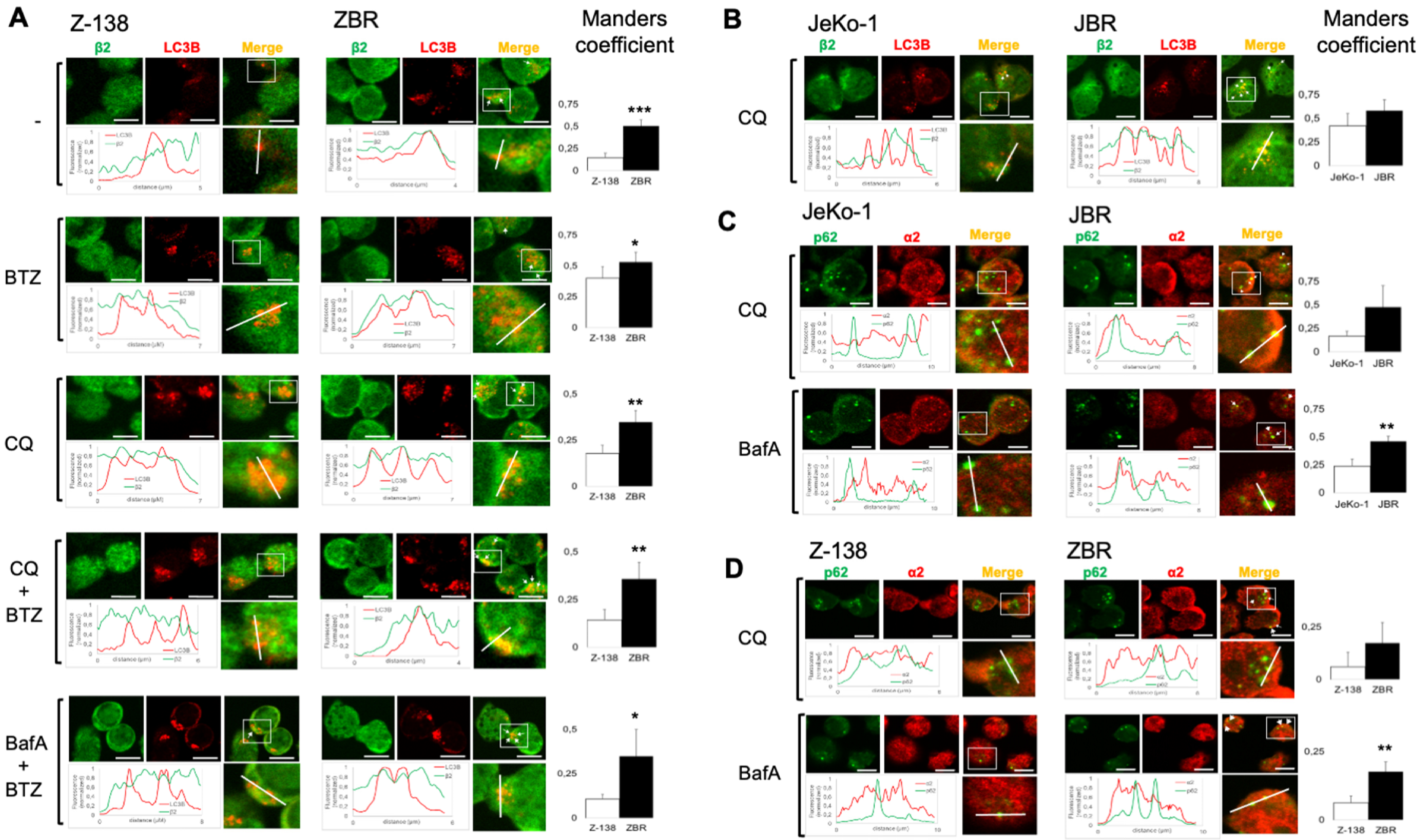
A
Z-138
ZBR
β2
LC3B
Merge
Manders coefficient
-
BTZ
CQ
CQ + BTZ
BafA + BTZ
Z-138
ZBR
B
JeKo-1
JBR
CQ
JeKo-1
JBR
C
p62
α2
BafA
D

Figure S5. Co-localization of proteasome subunits with autophagosomes in distinct MCL cell lines. (A and D) Z-138/ZBR and (B and C) JeKo-1/JBR were treated 8h with BafA 40 nM, CQ 40μM, BTZ 10nM, or with the indicated combined treatment. (A and B) Fixed cells were stained with anti-β2 antibody (green) and LC3B (red) or (C and D) with anti-α2 antibody (red) and anti-p62 (green). Images were analyzed by confocal microscopy, scale bar indicates 10 μM. Looking for co-localization of proteasome and autophagy proteins, normalized values of pixel intensity for both channels along the region indicated by the white lines were plotted. Colocalization were measured with Manders Correlation Coefficient (MCC) calculated in regions of interest (LC3B or p62 punctates). (n ≥20; mean ± SD; two-tailed Student's t-test, *p<0.05, **p<0.01, ***p<0.001).

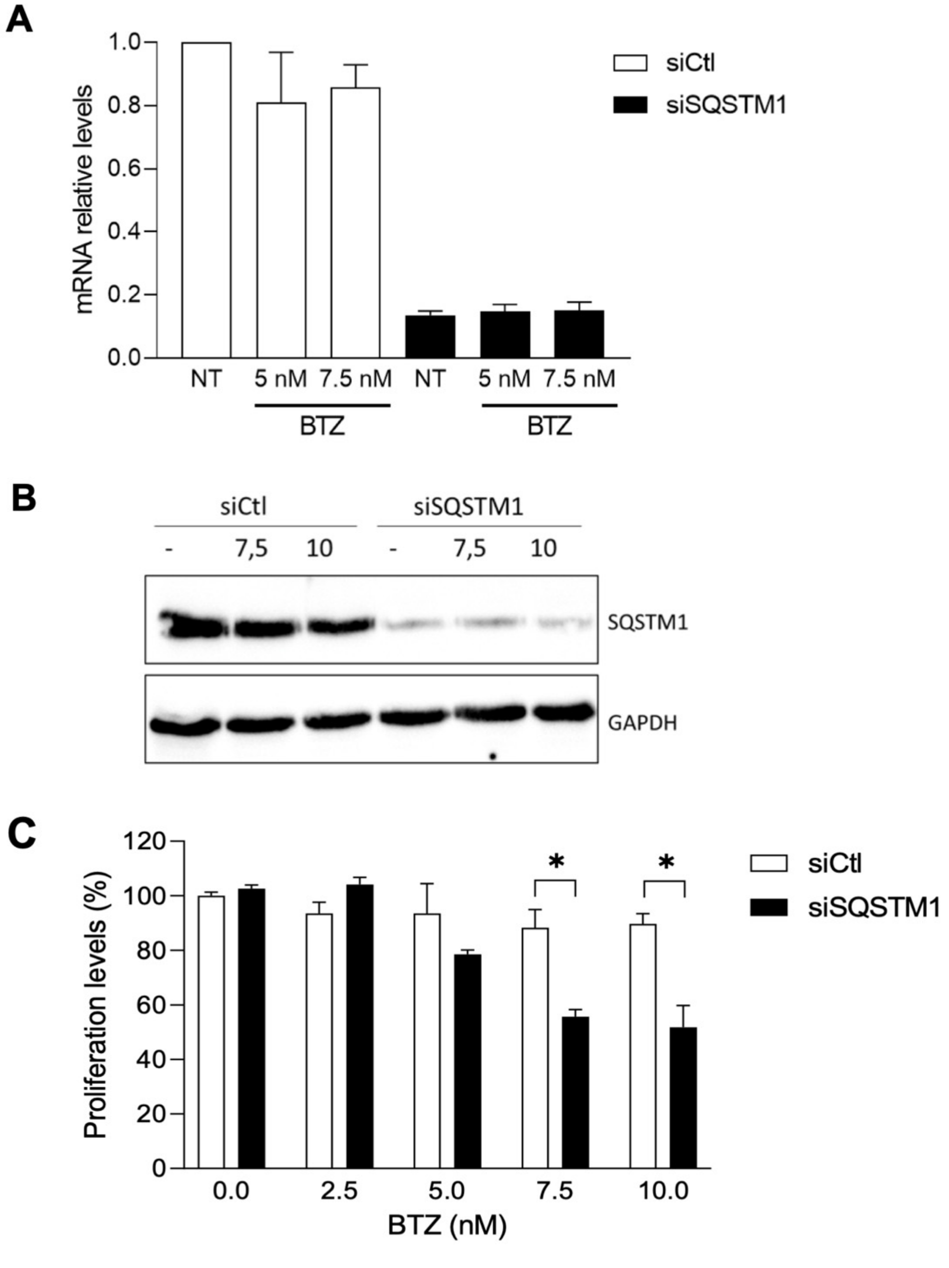

Figure S6. Silencing of p62 improves sensitivity to BTZ in BZT-resistant MCL cells. (A and B) ZBR cells were transfected with siRNA against SQSTM1 (siSQSTM1) or a non-targeted (scramble) sequence (siCtl) and treated 8 hours after transfection. (A) RNA and (B) protein were isolated after 24 hours of incubation with different

concentrations of BTZ. (C) Proliferation was determined by the Cell Titer-Glo Luminiscent Cell Viability Assay 24 hours after incubation with different concentrations of bortezomib. The IC50 were calculated in both siCtl (>50 nM) and siSQSTM1 (7.33 nM).
